# The research progress of biologics in elderly-onset rheumatoid arthritis (EORA)

**DOI:** 10.3389/fragi.2024.1511812

**Published:** 2025-01-23

**Authors:** Yujie Li, Yifan Liu, Yanhui Tian, Huimin Gu, Qingliang Meng, Jiakang Cui, Junfu Ma

**Affiliations:** ^1^ College of Orthopedics and Traumatology, Henan University of Chinese Medicine, Zhengzhou, China; ^2^ Department of Rheumatology, Henan Province Hospital of Traditional Chinese Medicine, Zhengzhou, Henan Province, China

**Keywords:** elderly-onset rheumatoid arthritis, biologic agents, infection, individualized treatment, review

## Abstract

*Elderly-onset rheumatoid arthritis (EORA)* is a distinct subtype of *rheumatoid arthritis* characterized by heightened treatment challenges due to immune aging and the complexity of comorbidities. This review systematically summarizes the definition, clinical features, epidemiological trends, therapeutic challenges, and the potential applications of biologic agents in *EORA*. It primarily focuses on the efficacy, safety, and individualized treatment strategies associated with various biologic agents. Studies indicate that biologics, such as *TNF-*α *inhibitors*, *IL-6 inhibitors*, and *JAK inhibitors*, can significantly reduce inflammation and improve joint function in *EORA* patients. However, their long-term use is closely linked to increased risks of infections, thrombosis, and malignancies, underscoring the importance of personalized treatment approaches and dynamic monitoring. Moreover, the advent of novel biologic agents, including *IL-17* and *IL-23 inhibitors*, as well as second-generation *JAK inhibitors*, offers additional therapeutic options for refractory patients and demonstrates substantial potential in optimizing both efficacy and safety. With the rapid progress of precision medicine and artificial intelligence (AI) technologies, gene profiling, biomarker analysis, and AI-assisted decision-making are gradually steering *EORA* treatment towards more personalized and precise strategies. However, the high cost of treatment and the limited accessibility of these technologies remain significant barriers in clinical practice. Future research should focus on validating the long-term safety of novel therapies and refining individualized treatment strategies to enhance patient outcomes and quality of life.

## 1 Introduction

**FIGURE 1:**
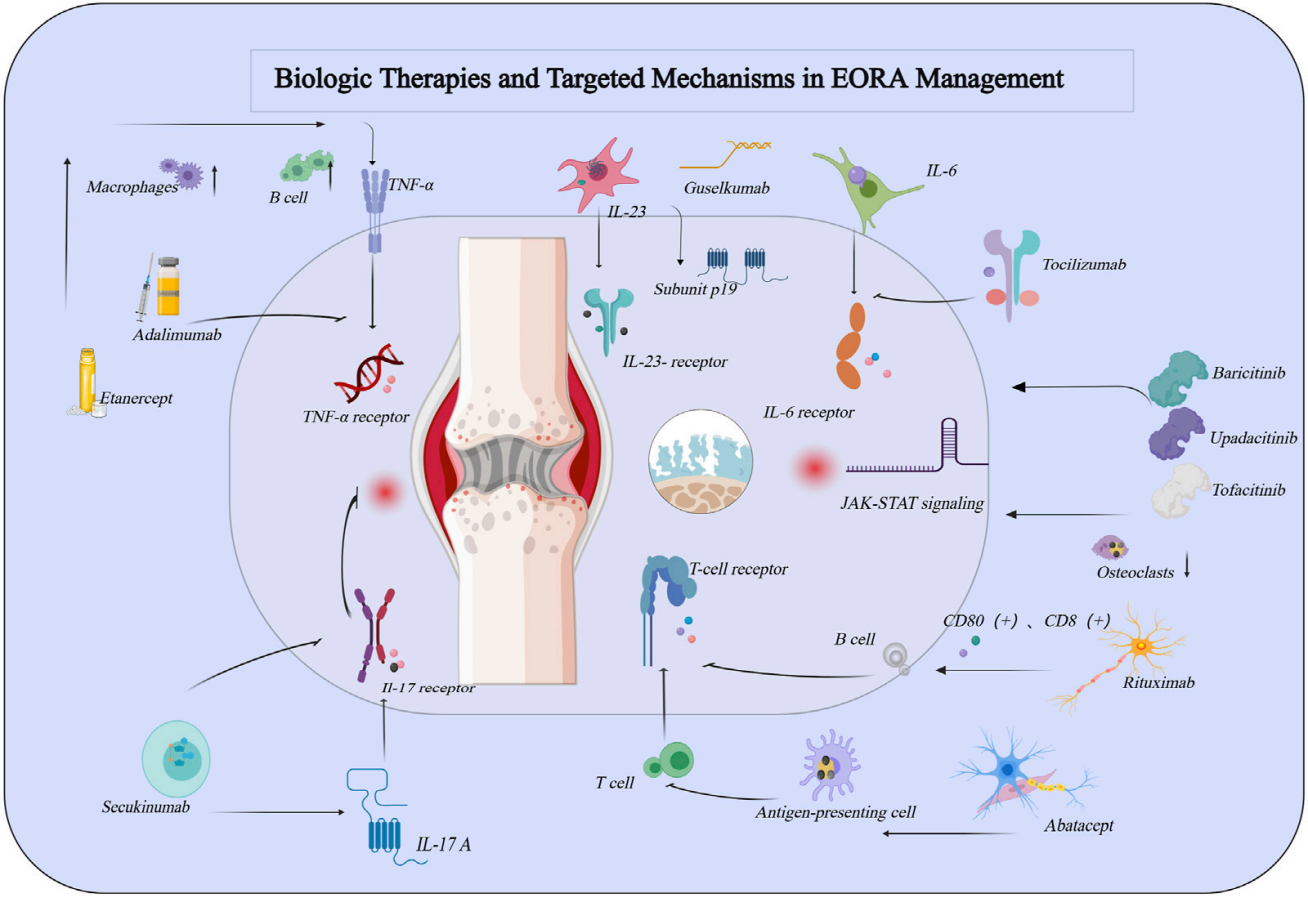
Schematic Representation of the Role of Common Biologic Agents in the Pathogenesis of Rheumatoid Arthritis. This figure was created using MedPeer (medpeer.cn).

With the accelerated aging of the global population, there has been increasing attention on *elderly-onset rheumatoid arthritis (EORA)*. As a distinct subtype of rheumatoid arthritis *(RA)*, EORA differs significantly from *young-onset rheumatoid arthritis (YORA)* in terms of pathophysiological mechanisms, clinical manifestations, and therapeutic needs. The physiological characteristics and chronic comorbidities of *EORA* patients make disease management more complex and increase treatment risks (Pavlov-Dolijanovic et al., 2023). Although biologic agents have brought new hope to the treatment of *RA*, research specifically targeting *EORA* patients remains limited, particularly in terms of the efficacy, safety, and individualized treatment strategies of different biologic agents (Baghdadi et al., 2015). Therefore, this review aims to summarize the current application of biologic agents in the treatment of *EORA*, focusing on their efficacy, safety, and individualized treatment strategies, while providing recommendations for optimizing clinical practice. Specifically, Table 1 summarizes the efficacy and safety data of various biologic agents in the treatment of *EORA*, offering crucial insights for clinical decision-making. As illustrated in Figure 1, the mechanisms of biologic agents in the treatment of elderly-onset rheumatoid arthritis are depicted, offering a comprehensive understanding of their therapeutic effects and underlying pathways.

**TABLE 1:** Summary of biologic therapy clinical applications in *EORA*.

Biologic agent type	Representative drugs	Target	Mechanism of action	Clinical benefits	Major risks	Indicated population
*TNF-*α *Inhibitors*	*Etanercept, Adalimumabb*	*TNF-*α	Blocks the binding of *TNF-*α to its receptor, reducing the release of inflammatory cytokines and delaying joint destruction	Improves disease activity and delays bone destruction	Infections, malignancies, injection site reactions	Patients with high inflammation and low infection risk
*IL-6 Inhibitors*	*Tocilizumab, Sarilumab*	*IL-6*	Blocks *IL-6* signaling pathways, inhibiting the release of inflammatory cytokines	Significantly effective for *EORA* patients with elevated *CRP* and *ESR*, good anti-inflammatory effect	Liver dysfunction, hyperlipidemia, high infection risk *(SAE* rate 20.2%)	*EORA* with high inflammatory activity and impaired immune function
*JAK inhibitors*	*Tofacitinib, Baricitinib, Upadacitinib*	*JAK-STAT* signaling pathway	Inhibit *JAK-STAT* pathway, alleviate inflammation	Rapid onset of action, improves morning stiffness and joint pain, suitable for *EORA* patients unresponsive to traditional therapies	Infection, thrombosis, liver dysfunction, hyperlipidemia	*EORA* with high disease activity, unresponsive to traditional treatments, and high quality-of-life demands
*IL-17 inhibitors*	*Secukinumab*	*IL-17*	Block *IL-17A*, inhibit the release of inflammatory factors	Control disease activity, suitable for patients who are unresponsive or intolerant to *TNF-*α inhibitors	Mild infections, injection site reactions	Patients who are unresponsive to or intolerant to *TNF-*α inhibitors
*IL-23* inhibitors	*Guselkumab*	*IL-23 (p19 subunit)*	Inhibit *IL-23* signaling pathway, suppress *Th17* cell differentiation	Great potential, but *EORA* efficacy not fully validated	Mild infections, injection site reactions	Further validation needed
*T-cell* Co-stimulation Inhibitors	*Abatacept*	*CD28/CD80, CD86*	Precisely inhibit *T-cell* activation, reduce immune response	Long-term efficacy, lower infection risk, long drug retention period	Higher discontinuation rate, suboptimal efficacy	*EORA* patients at high infection risk
*B-cell* Depletion Therapy	*Rituximab*	*“CD20+ B cells.”CD20*	Depletes *B cells*, reduces antibody-mediated immune responses	Controls disease activity, reduces immune-mediated inflammation, long-term remission in some patients	High infection risk, immunosuppressive-related adverse effects, potential malignancy risk	*EORA* patients who are refractory to other biologic therapies

1 Data sourced from multiple randomized controlled trials (RCTs) and real-world studies.

2 Abbreviations: *CRP* = C-reactive protein; *ESR*, erythrocyte sedimentation rate; *SAE*, serious adverse event.

## 2 Definition, epidemiology, and clinical features of *EORA*

*EORA* refers to rheumatoid arthritis *RA* that is first diagnosed in individuals aged 60 years or older. Compared to *YORA*, *EORA* exhibits significant differences in both pathological mechanisms and clinical manifestations, primarily due to the effects of immunosenescence and inflammaging. Immunosenescence refers to the gradual decline of immune function with age, leading to a reduced ability to fight infections and an increased susceptibility to autoimmune reactions, while inflammaging describes a state of chronic low-grade inflammation that results from the overexpression of pro-inflammatory factors, such as *TNF-*α and *IL-6*, in the elderly population. In *EORA* patients, inflammatory markers, such as *C-reactive protein (CRP)* and *erythrocyte sedimentation rate (ESR)*, are often elevated, and the disease tends to predominantly affect larger joints (e.g., the shoulders and knees), which exacerbates functional disability and significantly reduces quality of life (Targońska-Stępniak et al., 2021; Yoshii et al., 2020). The prevalence of *EORA* is estimated at approximately 1.5%–2% in the elderly population, with an increasing trend as individuals age. It is particularly common among females, especially postmenopausal women, in whom the decline in estrogen levels contributes to immune dysregulation, thereby increasing the risk of developing the disease (Tan et al., 2016; Turesson et al., 2015). Moreover, *EORA* patients frequently present with multiple chronic comorbidities, including *cardiovascular* diseases (e.g., *hypertension* and *coronary artery* disease) and *metabolic* disorders (e.g., *diabetes* and *hyperlipidemia*). These comorbidities complicate treatment regimens and require careful consideration of drug metabolism and safety (Jin et al., 2017; Vicente et al., 2021). In conclusion, the distinct pathological mechanisms and epidemiological characteristics of *EORA* highlight the need for personalized treatment strategies, which could provide new directions for future clinical research and therapeutic practice (Sato et al., 2021; Liu et al., 2024).

## 3 Application of biologic agents in *EORA*

In recent years, biologic agents have offered a diverse range of treatment options for patients with *EORA*. These medications target specific immune pathways, effectively inhibiting inflammatory responses, reducing disease activity, and delaying joint and bone damage (Dalal et al., 2019). However, due to the unique physiological characteristics and chronic comorbidities of *EORA* patients, the use of biologics presents an increased risk of adverse effects and poses additional management challenges. Currently, the biologic agents commonly used in clinical practice can be classified into seven major categories: *TNF-*α *inhibitors*, *IL-6 inhibitors*, *JAK inhibitors*, *B-cell* depletion therapies, *T-cell* co-stimulation blockers, *IL-17 inhibitors*, and *IL-23 inhibitors*. In the following sections, we will explore the mechanisms of action, efficacy, safety profiles, and reasons for discontinuation for each class of these biologics, providing a comprehensive guide to optimizing clinical treatment.

### 3.1 *TNF-*α *inhibitors*

#### 3.1.1 Adalimumab

*Adalimumab* is a fully human monoclonal antibody that specifically targets and blocks the binding of *TNF-*α to its receptors. This mechanism of action inhibits the release of pro-inflammatory cytokines, reduces systemic inflammation, and helps to slow the progression of cartilage and bone damage (Filippini et al., 2010). Due to its targeted action, *adalimumab* is considered a key therapeutic option for patients with *EORA*. Clinical studies demonstrate that in patients aged ≥65 years, *adalimumab* achieves a 38.8% improvement in *DAS28* scores, comparable to the 37.5% improvement observed in patients aged 18–65 years (*p* > 0.05). However, improvements in functional scores (*HAQ*) tend to be somewhat lower in elderly patients (see Table 1), which may be attributed to factors such as delayed treatment initiation, age-related immune system decline, and the greater burden of comorbidities (Veerasubramanian et al., 2024). While *adalimumab* is highly effective in halting bone damage and improving quality of life, its safety profile in elderly patients—especially those with compromised immune function—remains a significant concern. The most commonly reported adverse effects of *adalimumab* include an increased risk of infections, such as reactivation of tuberculosis and fungal infections, as well as malignancies, including skin cancer and lymphoma. These risks are particularly pronounced in older individuals who may already be immunosuppressed (Cometi et al., 2020). Studies indicate that the relative risk of severe infections in elderly patients treated with *TNF-*α *inhibitors* is 1.59 (95% *CI*: 1.45–1.76), with heightened risk in patients with underlying *cardiovascular* disease or a history of malignancy (Pavlov-Dolijanovic et al., 2023). Additionally, secondary treatment failure frequently occurs, with initial efficacy often diminishing over time, leading to eventual discontinuation of therapy. The discontinuation rate of *adalimumab* in elderly patients (21.8%) is significantly higher than in younger patients (16.9%, *p* < 0.05). This difference is primarily attributed to side effects, poor treatment tolerance, and the high costs associated with biologic therapies (Gossec et al., 2024). From a clinical management perspective, *adalimumab* is most suitable for patients with high disease activity but relatively low risks of infection and malignancy. For patients at an elevated risk of infection, alternative biologic agents such as *abatacept* or *tocilizumab* should be prioritized (Jiang et al., 2024; Choi et al., 2023). Given the potential for serious side effects, long-term monitoring of elderly patients on *adalimumab* therapy should focus on assessing disease activity (*DAS28, HAQ*), inflammatory markers (*CRP*, *ESR*), and any adverse events. Treatment regimens should be adjusted accordingly to balance efficacy and safety, with careful consideration given to dosing intervals and potential modifications based on individual patient response.

#### 3.1.2 Etanercept

*Etanercept* is a soluble tumor necrosis factor receptor fusion protein that binds competitively to *TNF-*α, thereby inhibiting its interaction with cell surface receptors. This mechanism effectively suppresses inflammation and delays joint and bone damage (Filippini et al., 2010). *Etanercept* has shown sustained efficacy in preventing bone destruction and improving inflammatory markers. Its relatively lower risk of infections provides a therapeutic advantage in elderly patients. However, some studies have reported suboptimal functional improvement (e.g., in *HAQ* scores) among elderly patients, which may be attributed to the burden of comorbidities and delayed treatment initiation. Regarding safety, the primary adverse effects associated with *etanercept* include infections (such as pneumonia and reactivation of tuberculosis) and injection site reactions. *Etanercept* is associated with a lower risk of infections than *adalimumab* (*OR* = 0.79, 95% *CI*: 0.46–1.34), and is generally better tolerated (D'Arcy et al., 2021). Nevertheless, *etanercept* may still be linked to an increased risk of skin cancer and lymphoma, requiring regular monitoring of the skin and lymphatic system in patients. The discontinuation rate of *etanercept* is primarily influenced by infection risks and injection site reactions, with the high cost of treatment also contributing to reduced patient adherence. Despite these challenges, *etanercept* typically demonstrates a higher drug retention rate in *EORA* patients compared to *adalimumab*, particularly in those with high disease activity but a lower risk of infections (Jiang et al., 2024). Overall, both *adalimumab* and *etanercept* are effective *TNF-*α *inhibitors* that improve *DAS28* scores and slow bone damage in *EORA* patients. However, they differ significantly in terms of safety profiles and patient suitability. *Adalimumab* carries a higher risk of infections and malignancies (see Table 1), making it more appropriate for patients with a low risk of infection. In contrast, *etanercept*, with its lower infection risk and better tolerability, is generally better suited for elderly patients with fewer comorbidities. Although injection site reactions are more common with *etanercept*, its overall tolerability is superior to that of *adalimumab*. Long-term follow-up should focus on dynamically monitoring disease activity (e.g., *DAS28* and *HAQ* scores), inflammatory markers, and adverse events. Treatment regimens should be adjusted based on individual patient responses to minimize the cumulative impact of potential side effects.

### 3.2 *IL-6* inhibitors

*Tocilizumab* is a monoclonal antibody that targets *interleukin-6* (*IL-6*), inhibiting the *IL-6* signaling pathway. This action reduces the release of pro-inflammatory cytokines, alleviating both joint inflammation and systemic inflammatory responses. Due to its targeted mechanism of action, *tocilizumab* is an important therapeutic option for *EORA* patients, particularly those with significantly elevated *CRP* and *ESR* levels who have not responded adequately to conventional *DMARDs*. A prospective study in Japan demonstrated that in patients aged ≥65 years, the improvement in the Clinical Disease Activity Index (*CDAI*) with *tocilizumab* was significantly higher than in younger patients (*p* = 0.03), confirming its superior efficacy in *EORA* (see Table 1) (Specker et al., 2022). However, despite its notable efficacy, the safety risks associated with *tocilizumab* should not be overlooked. In elderly patients, the incidence of serious adverse events (*SAEs*) is as high as 20.2%, which is significantly higher than in younger patients (11.5%, *p* < 0.0001). Common adverse events include bacterial pneumonia, fungal infections, liver dysfunction, and hyperlipidemia (Kondo et al., 2020). These risks are particularly pronounced in elderly patients with impaired immune function, underscoring the importance of individualized treatment strategies in *EORA*. Accordingly, comprehensive infection screening should be performed prior to treatment, and liver function, blood lipid levels, and other relevant parameters should be closely monitored during therapy to ensure the early detection and management of potential adverse effects. Liver dysfunction and infections are among the primary reasons for discontinuation of *tocilizumab*, highlighting the drug’s inherent risks and the need for flexible, personalized treatment adjustments. To optimize efficacy while minimizing risks, the dosing frequency and dosage of *tocilizumab* should be tailored to the patient’s immune status, comorbidities, and infection risks. For patients at higher risk of infections, a more conservative treatment approach should be considered, such as reducing the dosing frequency or lowering the dosage, while intensifying monitoring of treatment response and adverse events. Multidisciplinary management can help mitigate the adverse effects of treatment, extend drug retention, and improve long-term outcomes. Therefore, treatment decisions for *EORA* patients should not only be based on the drug’s efficacy but also take into account individual risks and treatment tolerance. This ensures that therapy remains both appropriate and safe. Furthermore, *Sarilumab,* another *IL-6 inhibitor*, has shown promise in reducing inflammation and improving joint function. However, clinical trial data specific to *EORA* patients is currently lacking, and further research is needed to assess its efficacy and safety in elderly populations.

### 3.3 *JAK* inhibitors

#### 3.3.1 Tofacitinib

*Tofacitinib* is an oral Janus kinase (*JAK*) *inhibitor* that modulates the *JAK-STAT* signaling pathway, effectively reducing both joint and systemic inflammation in patients with *EORA*. This mechanism makes it a valuable therapeutic option for patients who have not responded adequately to conventional *DMARDs* or *TNF-*α *inhibitors*. Clinical studies have shown that *tofacitinib* provides comparable improvements in *DAS28* scores in elderly patients as it does in younger individuals, demonstrating its effectiveness in disease control. However, concerns about its safety, especially in the elderly, have emerged. Compared to younger patients, elderly individuals (≥65 years) receiving *tofacitinib* have a significantly higher risk of serious adverse events (*SAEs*) — 17.56 per 100 patient-years versus 8.44 per 100 patient-years in younger cohorts. Additionally, elderly patients are at twice the risk of herpes zoster infections (6.40 vs 3.76 per 100 patient-years), and face elevated risks of venous thromboembolism and malignancy, particularly at the 10 mg dose. Notably, the risk of venous thromboembolism in this group is substantially increased, with a hazard ratio (*HR*) of 5.02 (95% *CI*: 1.44–17.47) (Shih et al., 2024). Given these risks, careful monitoring is essential when using *tofacitinib*, especially in elderly patients who typically have a reduced immune response and multiple comorbidities. The oral formulation of *tofacitinib* offers convenience, particularly for older patients who may struggle with adherence to injectable therapies. However, when compared to other *JAK* inhibitors, like *upadacitinib*, *tofacitinib* shows somewhat weaker effects in improving quality of life and reducing morning stiffness (Duran et al., 2022). Especially in patients with higher disease activity, clinical practice requires balancing effective treatment with monitoring for potential safety concerns. Adjustments to treatment, including dose modifications or closer monitoring for infections and thromboembolic events, may be necessary to ensure that the benefits of *tofacitinib* outweigh the risks (Wang et al., 2024). In summary, while *tofacitinib* represents a promising therapeutic option for *EORA* patients, its potential safety issues necessitate vigilant monitoring and individualized treatment strategies to mitigate risks while optimizing outcomes.

#### 3.3.2 Baricitinib

*Baricitinib* is a selective inhibitor of Janus kinases 1 (*JAK1*) and 2 (*JAK2*) that modulates the *JAK-STAT* signaling pathway, effectively reducing both joint and systemic inflammation in patients with *EORA*. It has demonstrated significant efficacy in patients with high disease activity, particularly in those who have not responded adequately to conventional *DMARDs*. Long-term studies show that both 2 mg and 4 mg doses of *baricitinib* consistently improve *DAS28* scores and *ACR20* response rates, with a notable advantage in controlling inflammation (Taylor et al., 2022). However, despite its strong therapeutic benefits, safety concerns with *baricitinib* should be carefully considered. In elderly patients, the incidence of serious infections is 5.5 cases per 100 patient-years, compared to 2.1 cases per 100 patient-years in younger patients. Pneumonia (0.6 cases per 100 patient-years) and herpes zoster (0.3 cases per 100 patient-years) are the most common infections observed. Additionally, there is an increased risk of venous thromboembolism (*VTE*), with deep vein thrombosis occurring at a rate of 0.49 cases per 100 patient-years and pulmonary embolism at 0.26 cases per 100 patient-years. The incidence of major adverse cardiovascular events (*MACE*) is 0.5 cases per 100 patient-years, highlighting the need for close monitoring of infection and thrombotic risks when using *baricitinib* (Keystone et al., 2015). Compared to *tofacitinib*, *baricitinib* carries a more pronounced risk of lipid abnormalities, especially in elderly patients who may require more frequent monitoring of lipid profiles. Despite these safety concerns, the rapid onset of action and stable long-term efficacy of *baricitinib* make it an important option for treating patients with high disease activity in *EORA*. In clinical practice, it is crucial to monitor not only infection risks but also lipid levels and cardiovascular health throughout treatment to ensure an optimal balance between efficacy and safety. *Baricitinib* is particularly well-suited for patients with higher quality-of-life demands, as it is especially effective in alleviating morning stiffness and joint pain (Genovese et al., 2016). This makes it complementary to other *JAK inhibitors*, such as *upadacitinib*, which may target different aspects of the disease. Ultimately, treatment with *baricitinib* requires individualized dosing (2 mg or 4 mg), tailored to each patient’s specific risk profile, along with regular monitoring of relevant blood parameters and infection indicators. By doing so, clinicians can maximize the therapeutic benefits of *baricitinib* while minimizing potential safety risks (Virtanen et al., 2024).

#### 3.3.3 Upadacitinib

*Upadacitinib* is a novel, selective *JAK1* inhibitor that, due to its high selectivity, significantly reduces the risk of off-target adverse effects. In multiple clinical trials, *upadacitinib* has shown a rapid onset of action and potent anti-inflammatory effects, particularly in improving pain, morning stiffness, and overall quality of life, outperforming *adalimumab*. The *SELECT-MONOTHERAPY* trial results showed that *upadacitinib* monotherapy significantly increased the *ACR20* response rate at 14 weeks (68% vs 41%, *p* < 0.001), with a corresponding improvement in *DAS28*-*CRP* scores (Kiełbowski et al., 2024). Furthermore, the *SELECT-EARLY* trial revealed that *upadacitinib* outperformed *methotrexate* (*MTX*) monotherapy in both efficacy and radiographic improvements, highlighting its potential as an early treatment option for *EORA* (Strand et al., 2021). Compared to *baricitinib*, *upadacitinib* shows superior efficacy in relieving morning stiffness and pain, along with significant improvements in both *ACR20* and *DAS28* scores. These factors make it a competitive option for patients with high disease activity in *EORA*. However, safety concerns with *upadacitinib* should not be overlooked. Its primary adverse events include infections (including upper respiratory infections and herpes zoster) and liver dysfunction (see Table 1). Research showns that the incidence of serious adverse events is 4.7%, with venous thromboembolism occurring in 0.6% of patients and liver function abnormalities in 0.8% (Fleischmann et al., 2019). While *upadacitinib* may present a slightly lower infection risk than *tofacitinib* and *baricitinib*, pneumonia and lipid abnormalities still require close monitoring, particularly in elderly patients. To minimize adverse reactions, reducing the dose from 30 mg to 15 mg and regularly monitoring liver function, blood lipids, and infection risks are recommended. This approach can help optimize long-term therapeutic efficacy while minimizing safety concerns (Smolen et al., 2019). Consequently, *upadacitinib* is particularly suited for *EORA* patients with higher quality-of-life demands, especially those seeking better control over morning stiffness and joint pain. However, clinical use should be individualized, with close monitoring to maintain a balance between efficacy and safety based on each patient’s specific risk profile.

### 3.4 *IL-17* inhibitors

#### 3.4.1 Secukinumab

*Secukinumab* is a fully humanized monoclonal antibody that targets *interleukin-17A* (*IL-17A*). It works by blocking the binding of *IL-17*A to its receptor, thereby inhibiting the release of pro-inflammatory cytokines and reducing joint and systemic inflammation in patients with *EORA*. In treating *EORA*, *secukinumab* not only effectively controls disease activity but also offers an important alternative for patients who do not respond to or cannot tolerate traditional *TNF-*α *inhibitors* (Sato et al., 2006). Clinical studies have consistently demonstrated *secukinumab*’s significant efficacy in treating EORA. For example, a Phase III trial showed that after 12 weeks of treatment, *secukinumab* significantly increased the *ACR20* response rate to over 60%, outperforming the placebo group. Further meta-analyses revealed that in patients with inadequate response to *TNF-*α (*TNF-IR*), *secukinumab* achieved *ACR50* and *ACR70* response rates that were 1.94 and 2.11 times higher than those of the placebo group, respectively (*p* < 0.01). Importantly, this efficacy was sustained over the long term, leading to substantial improvements in disease remission and functional recovery (Wu et al., 2019; Genovese et al., 2010). These findings highlight *secukinumab*’s potential not only in improving short-term symptoms but also in playing a critical role in long-term disease management strategies for *EORA* patients. In terms of safety, *secukinumab* has shown good tolerability, particularly concerning infection risk. The most common adverse events are mild infections (such as upper respiratory tract infections) and injection site reactions (see Table 1), while the incidence of severe infections remains relatively low—around 2.5%–3.0%, which is lower than the infection rates typically seen with *TNF-*α *inhibitors*. This relatively lower infection risk makes *secukinumab* an attractive option for elderly *EORA* patients (Kunwar et al., 2016; Chabaud et al., 1999). However, injection site reactions remain a frequent reason for discontinuation, highlighting the importance of targeted management and patient education to improve adherence and overall treatment experience. *Secukinumab*’s value in the treatment of *EORA* lies in its favorable balance between efficacy and safety. It is particularly well-suited for patients with high disease activity and a low infection risk. In clinical practice, selecting the appropriate patients and addressing injection site reactions can further enhance both the treatment’s effectiveness and patient satisfaction, making *secukinumab* a strong treatment option for *EORA* patients.

### 3.5 *IL-23* inhibitors

#### 3.5.1 Guselkumab

*Guselkumab* is a fully humanized monoclonal antibody that targets *interleukin-23* (*IL-23*) by binding to its *p19* subunit thereby inhibiting its signaling. This action prevents the differentiation of *Th17 cells* and the associated pro-inflammatory responses. *IL-23* activates *Th17 cells* through the *JAK-STAT* pathway, which contributes to the inflammatory processes and bone loss observed in rheumatoid arthritis (*RA*). Studies have shown that *IL-23* levels are significantly elevated in the serum and synovial fluid of *RA* patients, with the levels correlating closely with disease activity and bone erosion, supporting the potential of *IL-23* as a therapeutic target for *RA*. In terms of efficacy, *guselkumab* has demonstrated strong anti-inflammatory effects in other immune-mediated diseases, such as psoriasis and psoriatic arthritis. However, its clinical efficacy for the treatment of *EORA* remains inconclusive. A Phase II clinical trial showed *guselkumab* failed to significantly improve *DAS28* scores or meet primary efficacy endpoints, suggesting that its effectiveness in *RA* treatment requires further investigation. This suggests that, while *guselkumab* has shown promise in managing other immune-mediated diseases, its potential application in *EORA* still requires more robust clinical data to support its use (Yuan et al., 2019; Pastor-Fernández et al., 2020). From a safety perspective, *guselkumab* has generally shown good tolerability. The most common adverse events include mild infections (e.g., upper respiratory tract infections) and injection site reactions, while the incidence of severe infections is low, comparable to other biologic agents (see Table 1). However, since *IL-23* plays a crucial role in maintaining immune tolerance, long-term inhibition of *IL-23* may increase the risk of autoimmune diseases or infections. Therefore, regular monitoring of patients—especially those in the *EORA* population, who have a higher risk of infection—is critical during treatment. Although current clinical evidence does not fully support the widespread use of *guselkumab* in the treatment of EORA, its mechanism of inhibiting the *IL-23*/*IL-17 axis* offers a promising new therapeutic approach. As more clinical research and data accumulate, *guselkumab* may become an important therapeutic option for patients with *EORA*.

### 3.6 *T-cell* Co-stimulation inhibitors

#### 3.6.1 Abatacept

*Abatacept* is a *T-cell* co-stimulation inhibitor that works by selectively blocking *T-cell* activation, thereby reducing the transmission of inflammatory signals. It is particularly well-suited for *EORA*, who often have compromised immune systems and a higher risk of infections. As such, *abatacept* has become a key treatment option in the management of *EORA* (Nawrot et al., 2018). Clinical studies have shown that there is no significant difference in treatment outcomes between *RA* patients aged ≥65 years and those aged <65 years, with *abatacept* demonstrating comparable efficacy in both groups (Serhal et al., 2020). This suggests that *abatacept* offers stable and reliable efficacy in elderly patients, especially in those with impaired immune function. Additionally, *abatacept* has a notably higher drug retention rate compared to other biologics, with a median retention period of 254.1 weeks—significantly longer than that of TNF-α inhibitors (184 weeks) and JAK inhibitors (139.1 weeks). Its 2-year retention rate is 66.3%, further emphasizing its sustained effectiveness in long-term treatment (Rasch et al., 2003). These findings suggest that *abatacept* not only effectively controls disease activity in the short term but also provides long-lasting therapeutic benefits, which is particularly important for elderly patients who require ongoing treatment. Despite its strong efficacy and safety profile, inadequate response remains the primary reason for treatment discontinuation in elderly patients, with approximately 66.1% of patients stopping treatment due to insufficient effectiveness (Manfredi et al., 2024). Other factors such as smoking, joint erosion, and diabetes have also been identified as major risk factors for discontinuation (Sugihara, 2022). These considerations highlight the need for personalized treatment plans that take into account individual patient characteristics, in order to better manage disease progression and reduce the likelihood of premature discontinuation. Additionally, the economic burden of treatment can significantly affect adherence, particularly when the costs are high. Therefore, it is essential to factor in the patient’s immune status, comorbidities, and financial situation when developing a treatment strategy. Regular monitoring of disease activity and health status is also crucial to ensure timely adjustments to the treatment plan, maximizing efficacy while minimizing adverse effects (Ebina et al., 2019). With its low infection risk and long drug retention period, *abatacept* provides a safe and effective treatment option for elderly *EORA* patients, particularly those with compromised immune systems and a higher risk of infections. This positions it as one of the most clinically valuable therapeutic options for this patient population.

### 3.7 *B-cell* depletion therapy

#### 3.7.1 Rituximab

*Rituximab* is a monoclonal antibody that targets *CD20* on *B cells*, leading to their depletion and a subsequent reduction in antibody-mediated immune responses. This mechanism makes *rituximab* a particularly effective treatment option for *EORA* patients, especially those who have failed to respond to other biologic therapies. By inhibiting *B cell*-driven immune responses, *rituximab* not only controls disease activity but also helps modulate the overall immune system, offering a valuable alternative for *EORA* patients who are refractory to other treatments. While *rituximab* may not be as effective as certain other biologics, such as *tocilizumab*, in improving specific biomarkers like *PON1* activity, it has demonstrated significant benefits in controlling disease activity and alleviating immune-mediated inflammation. Clinical studies have shown that *rituximab* can effectively improve Disease Activity Score (*DAS28-CRP*) and Clinical Disease Activity Index (*CDAI*) scores, with some patients achieving long-term remission. These improvements are reflected in reduced joint inflammation, lower disease activity, and improved quality of life for patients (Razmjou et al., 2024). However, *rituximab* use carries significant safety risks, particularly regarding infections. It is crucial to closely monitor for early signs of bacterial and viral infections during treatment, with timely prophylactic measures being essential. Additionally, *rituximab*’s immunosuppressive effects may result in infusion-related reactions and other long-term adverse events related to immune suppression, including an increased risk of malignancies (see Table 1). Severe infections or poor drug tolerance are the primary reasons for discontinuation, underscoring the need for individualized patient management, particularly during the initiation and maintenance phases of treatment. In *EORA* patients, who often have naturally declining immune function, special attention should be given to monitoring immune status and infection risks to ensure the safety of the treatment. Despite the safety concerns, *rituximab*’s ability to control disease activity and reduce immune-mediated inflammation makes it an effective treatment option for many *EORA* patients, especially those who have not responded to other biologics (Pappas et al., 2014). To optimize its therapeutic benefits and minimize risks, clinical management should emphasize close monitoring, timely interventions, and careful patient selection. This approach will help ensure the most effective and safe use of *rituximab* in the treatment of *EORA.*

## 4 Clinical translation challenges of novel biologic therapies and the integration of precision medicine

Although novel biologic agents have demonstrated promising efficacy in clinical trials, their clinical translation faces several significant challenges. One of the most critical hurdles is integration of precision medicine into routine clinical practice, particularly in addressing patient heterogeneity, genetic backgrounds, and complex comorbidities. As the demand for personalized treatments grows, future therapeutic strategies will increasingly depend on selecting biologic agents based on a patient’s genetic profile, immune status, and disease characteristics, with the goal of achieving better and more predictable treatment responses. Precision medicine not only broadens the options for treating *EORA* but also lays the foundation for developing individualized treatment approaches. By combining genomics, artificial intelligence (AI), and clinical data, future treatments will be more personalized and accurate, allowing for better prediction of patient responses to therapies and potential adverse events. For example, AI models can incorporate a patient’s genetic information to predict the risks of complications such as thrombosis or infections, enabling timely adjustments to the treatment plan. This approach has the potential to significantly enhance treatment efficacy and improve patients’ quality of life (Mertz et al., 2023; Topol, 2019). However, despite the theoretical promise of novel biologic agents and precision medicine, high costs and technological barriers remain significant obstacles to their widespread application. The high cost of developing and deploying these biologic therapies, especially in low- and middle-income countries, limits access to these treatments. As a result, future research must focus on reducing the economic burden of these therapies, while promoting technological innovations to enhance accessibility, allowing more patients to benefit from these advances. In conclusion, technological innovation, supportive policies, and the ongoing development of precision medicine have the potential to improve the long-term prognosis of *EORA* patients and enhance their quality of life through the clinical use of novel biologic agents and personalized treatment strategies. Additionally, future treatments will focus not only on controlling disease but also on delivering personalized health management tailored to each patient’s needs.

## 5 Conclusion

As the global population ages, *EORA* has become a major focus in rheumatoid arthritis (*RA*) research. Recent advances in biologic therapies and precision medicine have significantly improved treatment options for *EORA* patients, especially those who fail to respond to traditional therapies. Novel biologic agents, such as *IL-17 inhibitors, IL-23 inhibitors*, second-generation *JAK inhibitors*, and *B-cell* depletion therapies, have shown strong efficacy in modulating immune responses. These treatments not only reduce disease activity and systemic inflammation but also mitigate long-term risks of infections, thrombosis, and malignancies.The integration of precision medicine has further advanced the treatment of *EORA*. By leveraging genetic profiling and artificial intelligence (AI)-assisted decision-making, clinicians can now tailor treatment plans to the unique needs of individual patients, enhancing both efficacy and safety. The identification of genetic markers like HLA-DRB1*04 and AI-based drug response predictions provides a solid foundation for personalized therapies. Despite the promising potential of novel biologic therapies and precision medicine, their widespread adoption faces significant challenges, particularly in low- and middle-income countries. Future research should focus on long-term safety, further validation of efficacy, and strategies to reduce treatment costs, ensuring broader patient access. With ongoing technological innovations and policy support, individualized treatment strategies have the potential to greatly improve long-term prognosis and quality of life for *EORA* patients. In summary, the combination of emerging biologic therapies and precision medicine provides an optimistic outlook for treating *EORA*. In the future, research should focus on overcoming the challenges of large-scale implementation, optimizing treatment protocols, and further improving patient outcomes and quality of life.
